# Integrating ultrasound technology into teaching gross anatomy: point of order!

**DOI:** 10.3402/meo.v18i0.20888

**Published:** 2013-06-07

**Authors:** Francis A. Fakoya

**Affiliations:** Department of Anatomical Sciences, School of Medicine, St. George’s University, Grenada, West Indies

Ultrasound (USS) or ultrasonic imaging is now a mature technology – having a well-established place in clinical practice and accounting for about one in four of all imaging procedures worldwide (1). More recently, USS has become the latest non-invasive method of morphological study to aid or supplement the teaching of gross human anatomy in some medical school curricula (2–6). Indeed, an expanding use of the technology – whether correct or incorrect, or by the trained or untrained – has caused the field of radiology to relax its hold on USS (7).

USS anatomy is based on its ability to reflect an image of the structures under view which, like any other skill, requires both practice and content-specific knowledge. The popular brightness-mode (B-mode) imaging involves transmitting small pulses of ultrasound echo from a transducer into the body. As the ultrasound waves penetrate body tissues of different acoustic impedances along the path of transmission, some are reflected back to the transducer (echo signals) while others penetrate deeper. The echo signals returned from many sequential pulses are subsequently processed and combined to generate an image. Thus, an ultrasound transducer works both as a speaker (generating sound waves) and a microphone (receiving sound waves) (8).

A detailed discussion of the underlying physics and scientific technologies is not warranted here. However, suffice it to say that while USS has much potential to aid in the teaching of medical students, numerous questions arise, including:

1. What do medical students need to know about USS to reliably resolve and interpret resulting anatomical images? For example, how much physics is required to understand (for example) the white, black, and gray hyper- and hypo-echoic zones displayed in images?
2. How much additional workload is involved – and when would this training take place? Do established resources on USS anatomy exist – such as guidebooks, journals, or web resources? Are there currently such courses/curricula available to bring medical students up to speed on USS – similar to the use of the microscope in microanatomy?
3. How much clinically-relevant benefit can medical students glean from using USS to learn gross anatomy? Will USS-based anatomy programs become more feasible now that equipment is portable, less expensive, and easier to use?

While the technology may have become more ‘user friendly’, interpreting the resulting images remains a mix of art and science. To illustrate, despite advances in ultrasound imaging technology, a number of inherent sources of ‘noise’ persist to compromise image quality and impair diagnostic utility (9). One such artifact is ‘speckle’, which arises from coherent wave interference and gives a granular appearance to an otherwise homogeneous region of tissue. Speckle reduces image contrast and detail resolution, and makes it difficult to identify abnormal tissue patterns (or texture) that may indicate disease. Other sources of noise include ‘clutter’ – spurious echoes often seen within structures of low echogenicity (e.g., cysts) or within amniotic fluid, and are often confused with ‘real’ targets.

All these confound the ability of the radiologist or sonographer to recognize tissue anomalies by interfering with what is, essentially, a pattern recognition process in the observer's brain (9). Used in gross anatomy, can a medical student (or instructor) navigate these subtleties to delineate normal from abnormal tissue topography?

It may be argued that if medical students can, with only minimal background in Electronmicroscopy (EM) processing and preparations, grasp ultra-structural images produced using Transmission Electronmicroscope (TEM) or Scanning Electronmicroscope (SEM), they should be able to understand USS images. Indeed, I would contend that a hybrid format whereby traditional dissection/prosection is integrated with USS-based content will enhance medical students’ comprehension of gross human anatomy. However, some introductory training would probably be required to aid students’ interpretations of USS images. This process could begin with using USS in a cadaver lab, and later proceed to live subjects (simulated patients or student peers) for more straightforward observational tasks (e.g., evaluating the recruitment of muscles, biofeedback, etc.) requiring a basic understanding of the main controls, safety issues, etc. Further training would be warranted when using USS to make objective measurements or to monitor structural changes. Experienced radiologists should be involved in setting the protocol for using USS equipment; standard images of different tissues and organs should also be provided, as well as outlets for troubleshooting.

Of course, to use the technology for instructional purposes, teachers interested in USS anatomy must themselves be trained in using the equipment. However, this may likely occur within current anatomy curricula that have been shortened (10). In addition, other radiological tools and approaches may well be required to corroborate anatomical findings and interpretations. While an in-depth understanding of how different conditions effect the ultrasound image – as well as how and where artifacts occur – is required for many diagnostic applications, the level of knowledge needed to correlate gross anatomical structures is perhaps best acquired via ‘physics of ultrasound’ courses offered in many sonographer or radiology tech training programs.

Medical students of the 21st century are a joystick generation; they quickly adopt and readily grasp most things ‘high tech’. With most USS tools emulating these configurations, combined with the widening availability of relevant resource materials, most medical students will need only minimal supervision and guidance to learning gross anatomy assisted by USS technology.
